# Best Practices in Addressing Multimorbidity Among Black Los Angeles Residents: Insights From the MOSAIC Initiative

**DOI:** 10.1093/geroni/igaf122.1825

**Published:** 2025-12-31

**Authors:** Angela Gutierrez, Kyeongwon Kim, Anna Lucas-Wright, Bo-Kyung Kim, Sonya Brooks, Courtney Thomas Tobin

**Affiliations:** Western University of Health Sciences, Pomona, California, United States; University of California, Los Angeles Fielding School of Public Health, Los Angeles, California, United States; Charles R. Drew University of Medicine and Science, Los Angeles, California, United States; University of California, Los Angeles Fielding School of Public Health, Los Angeles, California, United States; University of California, Los Angeles, Los Angeles, California, United States; University of California, Los Angeles Fielding School of Public Health, Los Angeles, California, United States

## Abstract

The M.O.S.A.I.C. (Multimorbidity Outcomes & Solutions for African/Black Americans in California) Initiative is a community-engaged research collaborative focused on understanding and addressing multimorbidity among Black communities. A key component of this initiative is developing effective health communication strategies to bridge the gap between research and real-world impact. Despite growing efforts to address persistent health disparities among Black Americans, there remains a critical need to identify effective health communication strategies for disseminating health information about multimorbidity to Black communities. This study aimed to identify best practices in health communication for developing and sharing multimorbidity health information for Black Los Angeles residents and to determine key components for inclusion in a virtual toolkit. Data were collected through a consensus-building session with a Community Advisory Board (CAB) comprising healthcare providers, organizational leaders, and community stakeholders with 20+ years of community health experience. Structured group discussions were followed by a consensus-building survey. The top-ranked health communication strategy was printed pamphlets, followed by in-person education sessions. A three-way tie emerged for third place: virtual presentations, social media posts, and videos. Social media and podcasts were preferred for young adults (18–29) and early midlife adults (30–44). Printed materials and in-person education were preferred for adults 45 and older. These best practices inform The MOSAIC Initiative’s efforts to develop tailored, community-driven health communication strategies for Black communities in Los Angeles. By aligning dissemination methods with age-specific preferences, these findings offer a foundation for more effective, culturally responsive multimorbidity interventions.

